# Association of Polymorphisms in *FSHR*, *ESR1*, and *BMP15* with Primary Ovarian Insufficiency and Meta-Analysis

**DOI:** 10.3390/diagnostics14171889

**Published:** 2024-08-28

**Authors:** Jeong Yong Lee, Young Ran Kim, Eun Ju Ko, Chang Soo Ryu, KyuBum Kwack, Eun Duc Na, Ji Eun Shin, Ji Hyang Kim, Eun Hee Ahn, Nam Keun Kim

**Affiliations:** 1 Department of Biomedical Science, College of Life Science, CHA University, Seongnam 13488, Republic of Korea; smilee3625@naver.com (J.Y.L.); ejko05@naver.com (E.J.K.); regis2040@nate.com (C.S.R.); kbkwack@cha.ac.kr (K.K.); 2 Department of Obstetrics and Gynecology, Fertility Center of CHA Bundang Medical Center, CHA University, Seongnam 13496, Republic of Korea; happyiran@cha.ac.kr (Y.R.K.); ned@chamc.co.kr (E.D.N.); 1219annie@chamc.co.kr (J.E.S.); bin0902@chamc.co.kr (J.H.K.)

**Keywords:** pregnancy, hormone, single nucleotide polymorphisms, SNP, genetics

## Abstract

Primary ovarian insufficiency (POI) can lead to menstrual disturbance, resulting in ovarian dysfunction before age 40. Prevalence of POI is usually less than 1%; however, ethnicity or population characteristics may affect prevalence. POI is a heterogeneous disease that results from abnormalities in immunological and hormonal factors. Genetic factors can also contribute to POI. Here, we examine *FSHR*, *ESR1*, and *BMP15* polymorphisms in patients with POI, and controls. We examined a hormonal gene that is important for pregnancy, follicle-stimulating hormone receptor (FSHR), as well as estrogen receptor 1 (ESR1), and associated it with FSHR expression, ovulation rate, and bone morphogenetic protein 15 (BMP15). We examined 139 Korean patients under age 40 with POI, and 350 Korean control participants without POI. Genotyping was performed by a polymerase chain reaction–restriction fragment length polymorphism (PCR–RFLP) and TaqMan assays. Each identified genotype was subjected to statistical analysis to determine the odds ratios (ORs) and 95% confidence intervals (CIs). In combination genotype analyses, *FSHR* rs6165 A > G combined with *ESR1* rs9340799 A > G, AG/GG (OR: 5.693; 95% CI: 1.088–29.792), as well as *FSHR* rs6166 A > G combined with *ESR1* rs9340799 C > T, AG/GG (OR: 5.940; 95% CI: 1.134–31.131), were significantly associated with POI prevalence. Furthermore, an *FSHR* rs6165 A > G and *BMP* rs17003221 C > T, AG/CC combination was associated with POI prevalence (OR: 1.874; 95% CI: (1.059–3.316; *p*-value: 0.031)). In meta-analysis, FSHR rs6165 AA vs. AG + GG is associated with POI (*p* = 0.0013), and ESR1 rs2234693 AA vs. AG + GG is also associated with POI (*p* = 0.0101). Here, we compared the genotypes of *FSHR*, *ESR1*, and *BMP15* in patients with POI, and controls. We found significant differences in genotype combinations between polymorphisms in *FSHR* and other genes. Through meta-analysis, we found that ESR1 rs9340799 and rs2234693 are associated with POI prevalence, and that BMP15 rs17003221 increases POI risk. These findings help to improve POI diagnosis in Korean women.

## 1. Introduction

Primary ovarian insufficiency (POI) is a menstrual disturbance resulting from ovarian dysfunction before age 40 [1,2]. Clinically, POI is characterized by amenorrhea or oligomenorrhea with raised gonadotrophins and low estradiol [1]. Prevalence of POI is usually less than 1%, although ethnicity or population characteristics may affect prevalence (Chinese: 0.5%; Japanese: 0.1%) [1,3,4]. POI diagnosis is determined by cessation of menstruation before age 40, an increase in follicle-stimulating hormone (FSH) levels (>40 mIU/mL in two consecutive measurements taken at least one month apart), and a decrease in estradiol (E2) (<15 pg/mL) [5].

Follicle-stimulating hormone receptor (FSHR) is an important hormone for maintaining pregnancy. *FSHR*, located in 2p21, contains ten exons; the first nine exons encode the extracellular domain, and exon ten encodes the C-terminal domain [6]. Given that FSH plays a critical role in ovarian function and follicle growth, FSHR dysfunction results in decreased folliculogenesis [7]. Further, several studies correlate *FSHR* mutations with POI diagnosis and various ovarian diseases [6,7,8].

E2 is also a very important factor for pregnancy initiation and maintenance. One important hormone receptor that functions in pregnancy is estrogen receptor 1 (*ESR1*). *ESR1* polymorphisms are associated with several diseases such as preeclampsia, breast cancer, obesity, and dysmenorrhea [9,10,11,12,13]. *ESR1*, located in 6q25, contains eight exons that encode the estrogen receptor alpha, which is a known ligand-dependent transcription factor that is important for hormone binding [10,13]. To date, several *ESR1* polymorphisms are associated with elevated E2 hormone levels [14,15].

As TGFβ super-family members, more than 20 different bone morphogenetic proteins (BMPs) have been identified, some of which are known to induce FSHR expression [16]. Specifically, *BMP15*, which is located on the X chromosome, is associated with infertility and increased ovulation rate [17]. Further, BMP15 and the paralog growth differentiation factor 9 (GDF9) play a crucial role in early folliculogenesis [18]. Previous reports found various variants of *BMP15* associated with POI occurrence [19]. Both genes promote the proliferation of granulosa cells; thus, variants in these genes are associated with cellular and molecular damage (i.e., a reduction in granulosa cells, defective granulosa cell secretion, and defective granulosa cell activity) [19]. *BMP15* variants also have a high incidence rate with POI diagnosis [20].

Single nucleotide polymorphisms (SNPs) are associated with various diseases, including POI [6,12,21,22]. In previous studies, especially for rs6165 and rs6166, FSHR is a highly controversial topic that is not associated with POI in Brazil, Argentina, Singapore, and New Zealand, or the Korean population [3,23,24,25,26]. For ESR1, rs9340799 and rs2234693 are reportedly associated with POI prevalence in the Iranian population [27]. However, in the Korean population, rs9340799 is not associated with POI prevalence [28]. Thus, we investigated these genes for polymorphisms found in Korean women. For this initial investigation of Korean women, we chose several SNPs (*FSHR*: rs6165 and rs6166; *ESR1*: rs9340799 and rs2234693; *BMP15*: rs17003221 and rs3810682) in our three genes of interest to examine.

## 2. Methods

### 2.1. Study Population

Blood samples were collected from 139 patients with POI (mean age ± SD: 31.8 ± 5.0 years), and 350 control participants without POI (mean age ± SD: 32.8 ± 3.7 years). All patients were diagnosed with POI, defined as cessation of menstruation before age 40, and they were given two serum FSH concentration measurements >40 IU/L at the Department of Obstetrics and Gynecology of the CHA Bundang Medical Center from March 1999 to February 2010. Patients with a history of pelvic surgery, radiation exposure, cancer, autoimmune disorder, or genetic syndromes were excluded from this study. The control group consisted of 350 subjects who had regular menstrual cycles and at least one live birth. The control group was recruited from the CHA Bundang Medical Center. All patients and controls were Korean.

### 2.2. Genotyping

DNA samples from patients with POI and control participants were extracted using the G-DEX blood extraction kit (iNtRON Biotechnology Inc., Seongnam, Republic of Korea). All polymorphisms were identified by a real-time polymerase chain reaction using the TaqMan SNP Genotyping Assay Kit (Applied Biosystems, Foster City, CA, USA). We randomly chose approximately 20% of the PCR assays to validate the real-time analysis using an ABI 3730XL DNA Analyzer (Applied Biosystems, Foster City, CA, USA). The concordance of the quality control samples was 100%.

### 2.3. Publication Search

We collected publications in Pubmed about FSHR, ESR1, and BMP15 mutations and POI interaction. The following combinations of key words were used: (“rs6165” and “POI”, “rs6166” and “POI”, “rs9340799” and “POI”, “rs2234693” and “POI”, “rs3810682” and “POI”, “rs17003221” and “POI”), (“POI” or “POF” or “primary ovarian insufficiency” or “premature ovarian failure”), and (“polymorphism” or “variant” or “mutation” or “genotype” or “single nucleotide polymorphism” or “SNP”). Review articles are excluded; original data with a human population and written in the English language are included.

### 2.4. Statistical Analysis

Genotype frequency differences between patients with POI and control participants were compared using logistic regression. Allele frequencies were calculated to investigate Hardy–Weinberg equilibrium (HWE) deviations. To examine the association between gene polymorphisms and POI prevalence, odds ratios (ORs) and 95% confidence intervals (CIs) were calculated using GraphPad Prism 4.0 (GraphPad Software, San Diego, CA, USA) and MedCalc version 12.1.4 (MedCalc Software bvba, Ostend, Belgium). Because the present study was a retrospectively designed case–control study and the disease incidence rate data were not available, the actual relative risk cannot be determined. We used the GraphPad Prism 4.0 and Med-Calc version 12.1.4 statistical programs. The sample size estimation with >80% statistical power, depending on expected ORs, was calculated using G*POWER3.0 (Institut für Psychologie, Christian-Albrechts-Universität Kiel, Kiel, Germany) [21].

## 3. Results

First, we examined the clinical profile of patients with POI and control participants, including age and hormone level. There are no differences between the age of the control participants and patients with POI (*p*-value: 0.100; mean age: 32.8 and 31.8 years, respectively, Table 1); however, when we examined hormone levels (FSH, LH, and E2) we found a significant difference between controls and patients (*p*-value < 0.0001; Table 1).

Next, we examined the genotype frequency in patients with POI and control participants. We examined six genetic loci in our three genes of interest, and found no significant differences between patients with POI and control groups (*ESR1* rs9340799 A > G, AA genotype, AOR: 1.194; 95% CI: 0.706–2.019; *p*-value: 0.509). Each group was in the Hardy–Weinberg equilibrium (Table 2). We also conducted an allele combination analysis on polymorphisms that are not associated with patients with POI (*FSHR* rs6165 A > G/*ESR1* rs2234693 T > C A-T, OR: 1.016; 95% CI: 0.653–1.521; *p*-value: 0.942) (Supplementary Table S1).

In the combined genotype analysis, *FSHR* rs6165 A > G combined with *ESR1* rs9340799 AG/GG was significantly different between patients with POI and control groups (AOR: 5.693; 95% CI: 1.088–29.792; *p*-value: 0.039; Table 3). *FSHR* rs6165 A > G combined with *BMP15* rs17003221 C > T was also significantly different in patients with POI compared to controls (AOR: 1.874; 95% CI: 1.059–3.316; *p*-value: 0.031; Table 3). When combined with *FSHR*, significant differences between the control and patient groups are shown to be risky (Table 3), while no significant protective effect of combined genotype analysis is present between patients and control (Supplementary Table S4).

We also conducted variance analyses between clinical parameters and gene polymorphisms. In the total participants (Supplementary Table S2), there is no difference between each SNP and clinical parameter (*FSHR* rs6165 A > G and FSH, *p*-value; 0.057). We observe an increasing correlation between LH level and *ESR1* rs9340799 A > G, while *ESR1* rs2234693 T > C shows a low correlation with LH levels. Patients with POI are not significantly different from control participants when comparing SNPs and clinical parameters (E2 level in *FSHR* rs6166 A > G; *p*-value: 0.397); however, in two *FSHR* variants (rs6165 A > G and rs6166 A > G), E2 levels show an increasing tendency (Table 4). FSH levels in *ESR1* rs9340799 A > G tend to increase, while the LH levels in *ESR1* rs2234693 T > C show a decreasing tendency (Table 4). Notably, in the control group, the only significant association is between *BMP15* rs71003221 C > T and levels of the hormone E2 (*p*-value: 0.038; Supplementary Table S3).

**Figure 1:**
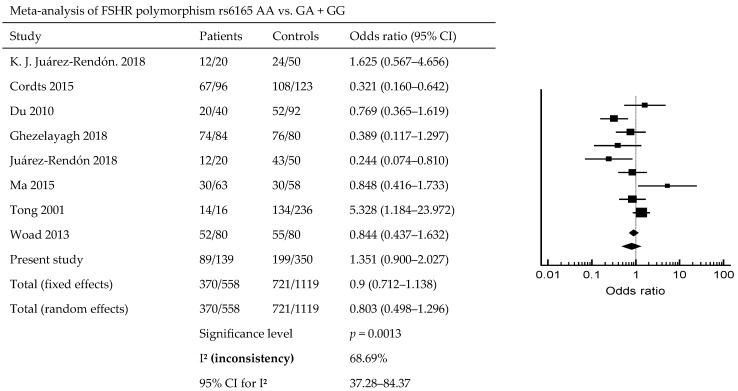
Forest plot of meta-analysis of FSHR polymorphisms. Meta-analysis of FSHR rs6165 AA vs. GA + GG in POI risk [3,8,23,25,29,30,31].

**Figure 2:**
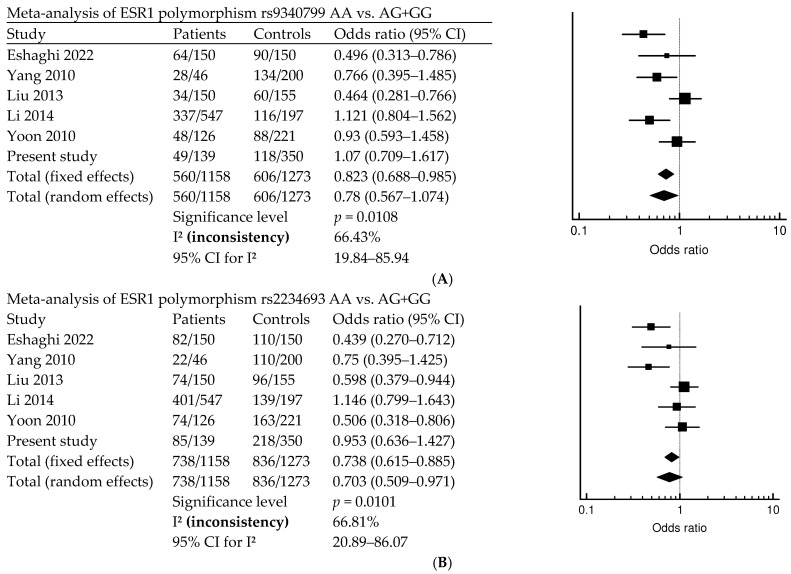
Forest plot of meta-analysis of ESR1 polymorphisms. Meta-analysis of rs9340799 AA vs. AG + GG (**A**) and rs2234693 AA vs. AG + GG (**B**) in POI risk [27,28,32,33,34].

**Figure 3:**
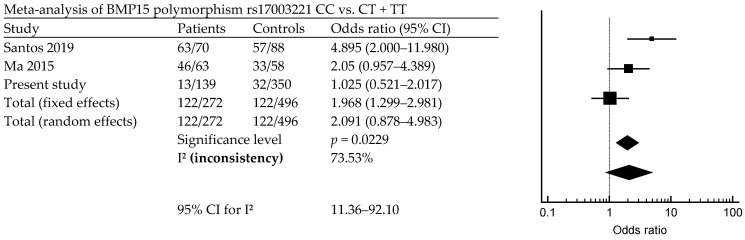
Forest plot of meta-analysis of BMP15 polymorphisms. Meta-analysis of rs17003221 CC vs. CT + TT in POI risk [31,35].

The results of the meta-analysis found that there is a significant association between POI risk and FSHR1 rs6165. A previous study, and our study—which contains a total of 558 patients and 1119 controls—shows that FSHR1 rs6165 is associated with POI (*p* = 0.0013; OR = 0.9; 95% CI, 0.712–1.138) (Figure 1). Figure 2 indicates that ESR1 rs9340799 AA vs. AG + GG is associated with POI risk (*p* = 0.0108; OR = 0.823; 95% CI, 0.688–0.985). Meta-analysis of BMP15, rs17003221 CC vs. CT + TT, with a total of 272 patients and 496 controls, is associated with POI risk (*p* = 0.0229; OR = 1.968; 95% CI, 0.878–4.983) (Figure 3).

## 4. Discussion

We have found hormonal gene polymorphism associations between patients with POI and control participants. In these analyses, we investigated six polymorphisms in three genes (*FSHR*, *BMP15*, and *ESR1*) and showed how they correlated with patients or controls. FSHR is essential for follicle growth and ovulation [16]. As the expression of FSHR increases, follicles grow; if FSHR decreases, follicles are degraded via follicular atresia [36]. In our genotype analysis, *FSHR* rs6165 polymorphisms were not associated with patients with POI, and ANOVA found that no genotype had a significant correlation with hormone levels (E2, FSH, LH).

In 2018, Juárez-Rendón et al. evaluated *FSHR* rs6165 in Mexican females and found no significant differences between patients with POI and controls [8]. In our previous study, *FSHR* rs6165 A > G was associated with recurrent implantation failure [37]. In a previous report, *FSHR* rs6166 variants showed no differences between patients with POI and control participants [26]. Furthermore, in the Chinese Han population, there are no significant associations between patients with POI and control participants [38]. However, other *FSHR* variants, rs1394205 and rs140106399, are significantly associated with POI in this population [38]. Another study in an Asian subgroup reported *FSHR* rs6166 as a risk for patients with POI in both a fixed-effect model and a random-effect model [7]. Here, we investigated the correlation between hormone levels and genotype correlation, but found no differences. Likewise, Neves, A.R. et al. also reported no statistical difference in *FSHR* variants and E2 levels [39].

More than 20 BMPs have been identified, some of which are known to induce *FSHR* expression [40]. Of them, BMP15 is known to regulate follicle development, oocyte quality [8], and to increase mRNA expression in the SMAD and p38 MAPK pathways, which are important in granulosa cells [16]. *BMP15* rs17003221 is previously reported to be associated with Brazilian patients with POI; however, in this study, a reason for this is not shown [35]. Further, *BMP15* rs3810682 was not significantly different between patients with POI and the control group. Previous reports found that the *BMP15* heterozygous mutation Y235C is associated with hypergonadotropic ovarian failure, and that various variants are associated with POI prevalence [21,37,41].

ESR1 plays an essential role in ovarian follicle growth, as estrogen receptor deficiencies result in fertility issues due to abnormal folliculogenesis [2,42]. Variations in ESR1 are associated with elevated levels of the E2 hormone. In our study, we examined two ESR1 polymorphisms, rs9340799 and rs2234693, but found no significant differences in genotype analysis. A study also previously reported no significant differences between the rs1569788 intron variant in Korean patients with POI and controls [43]. However, in Iranian patients with POI, both rs9340799 and rs2234693 were significantly different from control participants [27]. ESR1 is a target of the alpha-lipoic-acid (ALA) pathway, which is a recently reported treatment of POI [44]. Additionally, we found that meta-analysis of rs6165, rs9340799, rs2234693, and rs17003221 is associated with POI risk (Figure 1, Figure 2 and Figure 3). FSHR1 rs6166 and BMP15 rs3810682 are not associated with POI risk. As shown by the meta-analysis, not all studies are significantly different; however, results from the meta-analysis have found that FSHR rs6165 AA vs. GA + GG is associated with POI occurrence, but the other locus, rs6166, is not associated with POI occurrence (*p* = 0.0013). In a meta-analysis, no significant differences between patients with POI and controls were found for *FSHR* rs6165 and rs6166 in the overall analyses (sample size; case/control; rs6165, 590/1170; rs6166, 640/1333) [7]. ESR1 gene loci rs9340799 AA vs. AG + GG and rs2234693 AA vs. AG + GG are associated with POI (rs9340799, *p* = 0.0108; rs2234693, *p* = 0.0101). Not all of the SNPs are associated with POI in our studies, and while many studies are not associated with POI, there are differences in the meta-analysis [32]. BMP15 only has a significant difference in rs17003221 CC vs. CT + TT (*p* = 0.0229) rs3810682, and does not have a different meaning between the POI.

There are several limitations to our study, including a small sample size for both patients and controls. Further, we only examined genetic variants in the Korean population. We examined *FSHR*, *ESR1*, and *BMP15* SNPs and did not find a clear influence on POI. Additionally, the mechanism by which these genes function in POI is unclear; therefore, confirmation in vitro and in vivo is necessary. Given that our study was limited to the Korean population, additional large-scale studies in other ethnic populations are needed.

## Figures and Tables

**Table 1:** Comparison of clinical profiles between women with POI and controls.

Characteristics	Controls (*n* = 350)	POI (*n* = 139)	*p*
Age (years)	32.8 ± 3.7	31.8 ± 5.0	0.100 ^a^
Live births	1.59 ± 0.57	-	N/A
Average gestational age (weeks)	39.24 ± 1.47	-	N/A
FSH (mIU/mL)	8.1 ± 2.9	58.6 ± 23.7	<0.0001 ^b^
LH (mIU/mL)	3.3 ± 1.8	25.7 ± 16.3	<0.0001 ^b^
E2 (pg/mL)	26.1 ± 14.3	28.8 ± 73.5	<0.0001 ^b^

Note: POI, primary ovarian insufficiency; FSH, follicle-stimulating hormone; LH, luteinizing hormone; E2, estradiol; N/A, not applicable. ^a^ *t*-test, ^b^ Mann–Whitney test.

**Table 2:** Comparison of genotype frequencies of *BMP15*, *ESR1*, and *FSHR* polymorphisms between the POI and control subjects.

Genotypes	Controls (*n* = 350)	POI (*n* = 139)	COR (95% CI)	*p*	AOR (95% CI)	*p*
*FSHR* rs6165 A > G						
AA	151 (43.1)	50 (36.0)	1.000 (reference)		1.000 (reference)	
AG	147 (42.0)	70 (50.4)	1.438 (0.937–2.207)	0.096	1.546 (0.910–2.627)	0.108
GG	52 (14.9)	19 (13.7)	1.104 (0.597–2.041)	0.754	1.396 (0.618–3.151)	0.422
Dominant (AA vs. AG + AA)			1.351 (0.900–2.027)	0.147	1.467 (0.883–2.435)	0.139
Recessive (AA + AG vs. AA)			0.907 (0.515–1.599)	0.737	0.958 (0.473–1.942)	0.906
HWE-*P*	0.104	0.480				
*FSHR* rs6166 A > G						
AA	156 (44.6)	52 (37.4)	1.000 (reference)		1.000 (reference)	
AG	143 (40.9)	69 (49.6)	1.448 (0.946–2.215)	0.088	1.677 (0.989–2.844)	0.055
GG	51 (14.6)	18 (12.9)	1.059 (0.568–1.973)	0.857	1.293 (0.561–2.978)	0.547
Dominant (AA vs. AG + AA)			1.345 (0.899–2.013)	0.149	1.547 (0.932–2.567)	0.092
Recessive (AA + AG vs. AA)			0.872 (0.490–1.554)	0.642	0.873 (0.420–1.813)	0.716
HWE-*P*	0.056	0.509				
*ESR1* rs9340799 A > G						
AA	232 (66.3)	90 (64.7)	1.000 (reference)		1.000 (reference)	
AG	105 (30.0)	43 (30.9)	1.056 (0.687–1.623)	0.805	1.194 (0.706–2.019)	0.509
GG	13 (3.7)	6 (4.3)	1.190 (0.439–3.226)	0.733	1.583 (0.489–5.125)	0.443
Dominant (AA vs. AG + GG)			1.070 (0.709–1.617)	0.746	1.231 (0.745–2.034)	0.418
Recessive (AA + AG vs. GG)			1.170 (0.435–3.141)	0.756	1.467 (0.461–4.665)	0.516
HWE-*P*	0.794	0.765				
*ESR1* rs2234693 T > C						
TT	132 (37.7)	54 (38.8)	1.000 (reference)		1.000 (reference)	
TC	163 (46.6)	63 (45.3)	0.945 (0.615–1.452)	0.796	0.938 (0.553–1.590)	0.812
CC	55 (15.7)	22 (15.8)	0.978 (0.544–1.759)	0.940	1.030 (0.485–2.186)	0.939
Dominant (TT vs. TC + CC)			0.953 (0.637–1.427)	0.816	0.943 (0.573–1.551)	0.817
Recessive (TT + TC vs. CC)			1.009 (0.588–1.729)	0.975	0.994 (0.502–1.968)	0.986
HWE-*P*	0.692	0.614				
*BMP15* rs17003221 C > T						
CC	318 (90.9)	126 (90.6)	1.000 (reference)		1.000 (reference)	
CT	32 (9.1)	13 (9.4)	1.025 (0.521–2.018)	0.942	1.083 (0.477–2.456)	0.849
TT	0 (0.0)	0 (0.0)	N/A	N/A	N/A	N/A
Dominant (CC vs. CT + TT)			1.025 (0.521–2.018)	0.942	1.083 (0.477–2.456)	0.849
Recessive (CC + CT vs. TT)			N/A	N/A	N/A	N/A
HWE-*P*	0.372	0.563				
*BMP15* rs3810682 C > G						
CC	336 (96.0)	134 (96.4)	1.000 (reference)		1.000 (reference)	
CG	14 (4.0)	5 (3.6)	0.896 (0.316–2.535)	0.835	1.315 (0.419–4.129)	0.639
GG	0 (0.0)	0 (0.0)	N/A	N/A	N/A	N/A
Dominant (CC vs. CG + GG)			0.896 (0.316–2.535)	0.835	1.315 (0.419–4.129)	0.639
Recessive (CC + CG vs. GG)			N/A	N/A	N/A	N/A
HWE-*P*	0.703	0.829				

Note: AOR was adjusted by age. COR, crude odds ratio; AOR, adjusted odds ratio; 95% CI, 95% confidence interval; HWE, Hardy–Weinberg equilibrium.

**Table 3:** Combined genotype analysis for the polymorphisms in POI patients and controls.

Genotype Combinations	Controls (*n* = 350)	POI Patients (*n* = 139)	AOR	*p*
FSHR rs6165 A > G/ESR1 rs9340799 A > G				
AA/AA	103(29.4)	28(20.1)	1.000 (reference)	
AA/AG	42(12.0)	20(14.4)	1.845 (0.765–4.451)	0.173
AG/AA	97(27.7)	48(34.5)	1.957 (0.983–3.895)	0.056
AG/AG	46(13.1)	18(12.9)	1.717 (0.725–4.063)	0.219
AG/GG	4(1.1)	4(2.9)	5.693 (1.088–29.792)	0.039
GG/AA	32(9.1)	14(10.1)	2.163 (0.721–6.485)	0.169
FSHR rs6165 A > G/BMP15 rs17003221 C > T				
AA/CC	135(38.6)	44(31.7)	1.000 (reference)	
AA/CT	16(4.6)	6(4.3)	2.159 (0.718–6.493)	0.171
AG/CC	134(38.3)	65(46.8)	1.874 (1.059–3.316)	0.031
GG/CC	49(14.0)	17(12.2)	1.552 (0.649–3.711)	0.323
FSHR rs6166 A > G/ESR1 rs9340799 A > G				
AA/AA	108(30.9)	28(20.1)	1.000 (reference)	
AA/AG	42(12.0)	22(15.8)	1.915 (0.794–4.617)	0.148
AG/AA	94(26.9)	48(34.5)	2.106 (1.058–4.190)	0.034
AG/AG	45(12.9)	17(12.2)	2.007 (0.861–4.676)	0.107
AG/GG	4(1.1)	4(2.9)	5.940 (1.134–31.131)	0.035
GG/AA	30(8.6)	14(10.1)	2.336 (0.778–7.018)	0.131
FSHR rs6166 A > G/BMP15 rs17003221 C > T				
AA/CC	140(40.0)	46(33.1)	1.000 (reference)	
AA/CT	16(4.6)	6(4.3)	2.223 (0.739–6.688)	0.155
AG/CC	130(37.1)	64(46.0)	2.047 (1.159–3.616)	0.014
GG/CC	48(13.7)	16(11.5)	1.423 (0.582–3.479)	0.439
FSHR rs6166 A > G/BMP15 rs3810682 C > G				
AA/CC	153(43.7)	50(36.0)	1.000 (reference)	
AA/CG	3(0.9)	2(1.4)	6.727 (0.917–49.325)	0.061
AG/CC	134(38.3)	66(47.5)	1.807 (1.048–3.114)	0.033
GG/CC	49(14.0)	18(12.9)	1.402 (0.604–3.252)	0.432

Note: AOR was adjusted by age. AOR, adjusted odds ratio; 95% CI, 95% confidence interval.

**Table 4:** Differences of various clinical parameters according to gene polymorphisms in POI patients.

Genotypes	FSH	LH	E2
	Mean ± SD	Mean ± SD	Mean ± SD
*FSHR* rs6165 A > G			
AA	58.719 ± 25.782	32.698 ± 25.430	18.529 ± 25.656
AG	58.019 ± 21.629	23.189 ± 11.891	22.662 ± 29.032
GG	60.242 ± 28.080	23.850 ± 9.496	56.346 ± 151.595
*P*	0.962	0.141	0.357
*FSHR* rs6166 A > G			
AA	59.221 ± 24.611	31.717 ± 24.144	20.881 ± 27.650
AG	57.663 ± 22.179	23.149 ± 12.165	21.439 ± 28.218
GG	60.242 ± 28.080	23.850 ± 9.496	56.346 ± 151.595
*P*	0.940	0.184	0.397
*ESR1* rs9340799 A > G			
AA	54.802 ± 25.919	21.567 ± 10.096	31.171 ± 89.059
AG	63.673 ± 17.406	33.267 ± 23.095	19.193 ± 25.048
GG	77.300 ± 18.584	27.100 ± 8.062	66.350 ± 50.417
*P*	0.135	0.663	0.669
*ESR1* rs2234693 T > C			
TT	56.900 ± 26.159	33.989 ± 29.351	32.313 ± 34.476
TC	61.706 ± 21.145	24.982 ± 12.299	17.105 ± 21.664
CC	54.920 ± 26.314	21.774 ± 8.317	43.461 ± 119.624
*P*	0.556	0.219	0.514
*BMP15* rs3810682 C > G			
CC	58.13 ± 23.634	25.797 ± 16.628	29.208 ± 74.203
CG	69.133 ± 27.710	24.4 ± 10.623	8 ± 0.000
GG	N/A	N/A	N/A
*P*	0.436	0.886	0.778
*BMP15* rs17003221 C > T			
CC	58.646 ± 24.370	25.814 ± 16.721	28.071 ± 75.044
CT	58 ± 7.708	24.057 ± 4.438	40.333 ± 50.644
TT	N/A	N/A	N/A
*P*	0.958	0.857	0.782

Note: FSH, follicle-stimulating hormone; LH, luteinizing hormone; E2, estradiol; N/A, not applicable.

## Data Availability

The data presented in this study are available on request from the corresponding author. The data are not publicly available due to another publication and personal information, but are available from the corresponding author on reasonable request.

## Supplementary Materials

The following supporting information can be downloaded at: https://www.mdpi.com/article/10.3390/diagnostics14171889/s1, Figure S1. Forest plot of meta-analysis of FSHR polymorphisms. Meta-analysis of FSHR rs6166 AA vs. GA+GG in POI risk. Figure S2. Forest plot of meta-analysis of ESR1 polymorphisms. Meta-analysis of rs9340799 AA vs. AG+GG (A) and rs2234693 AA vs. AG+GG (B) in POI risk. Table S1. Allele combination analysis of *ESR1*, *FSHR*, and *BMP15* polymorphisms in POI and controls subjects by MDR. Table S2. Differences of various clinical parameters according to gene polymorphisms in total participants. Table S3. Differences of various clinical parameters according to gene polymorphisms in control subjects. Table S4. Combined genotype analysis for the polymorphisms in POI patients and controls.
