# Supplementary material for: Association of Polymorphisms in FSHR, ESR1, and BMP15 with Primary Ovarian Insufficiency and Meta-Analysis
Source: Diagnostics (Basel). 2024 Aug 28;14(17):1889. doi: 10.3390/diagnostics14171889 (PMC11393966; doi:10.3390/diagnostics14171889)
Supplement: Supplementary file 1 [file diagnostics-14-01889-s001.zip › Supplementary Tables.pdf]

Supplementary Table S1. Allele combination analysis of *ESR1*, *FSHR*, and *BMP15* polymorphisms in POI and controls subjects by MDR

Allele combination	Control (2n=700)	Case (2n=278)	OR (95%CI)	*P*
FSHR rs6165 A>G /FSHR rs6166 A>G				
A-A	0.8200	0.8058	1.000 (reference)	
A-G	0.0007	0.0000	N/A	N/A
G-A	0.0050	0.0054	1.281 (0.233-7.047)	0.676
G-G	0.1743	0.1888	1.092 (0.762-1.565)	0.631
FSHR rs6165 A>G /ESR1 rs9340799 A>G				
A-A	0.7704	0.7416	1.000 (reference)	
A-G	0.0503	0.0642	1.346 (0.745-2.43)	0.323
G-A	0.1360	0.1595	1.212 (0.819-1.793)	0.336
G-G	0.0433	0.0348	0.872 (0.419-1.816)	0.715
FSHR rs6165 A>G /ESR1 rs2234693 T>C				
A-T	0.7050	0.6911	1.000 (reference)	
A-C	0.1157	0.1146	1.016 (0.653-1.581)	0.942
G-T	0.1000	0.1164	1.176 (0.75-1.845)	0.48
G-C	0.0793	0.0778	1.011 (0.601-1.701)	0.968
FSHR rs6165 A>G /BMP15 rs17003221 C>T				
A-C	0.8062	0.7917	1.000 (reference)	
A-T	0.0145	0.0141	1.025 (0.318-3.305)	1.000
G-C	0.1709	0.1849	1.090 (0.758-1.566)	0.643
G-T	0.0084	0.0093	1.282 (0.318-5.172)	0.717
FSHR rs6165 A>G /BMP15 rs3810682 C>G				
A-C	0.8180	0.8010	1.000 (reference)	
A-G	0.0027	0.0047	1.285 (0.116-14.25)	1.000
G-C	0.1720	0.1900	1.135 (0.793-1.624)	0.489
G-G	0.0073	0.0043	0.514 (0.06-4.426)	1.000
FSHR rs6166 A>G /ESR1 rs9340799 A>G				
A-A	0.7751	0.7425	1.000 (reference)	
A-G	0.0499	0.0686	1.431 (0.8-2.559)	0.225
G-A	0.1313	0.1585	1.261 (0.851-1.869)	0.248
G-G	0.0437	0.0303	0.680 (0.308-1.505)	0.339
FSHR rs6166 A>G /ESR1 rs2234693 T>C				
A-T	0.7084	0.6897	1.000 (reference)	
A-C	0.1166	0.1215	1.071 (0.695-1.652)	0.756
G-T	0.0966	0.1179	1.254 (0.801-1.962)	0.322
G-C	0.0784	0.0710	0.939 (0.548-1.609)	0.820
FSHR rs6166 A>G /BMP15 rs17003221 C>T				
A-C	0.8106	0.7972	1.000 (reference)	
A-T	0.0144	0.0139	1.022 (0.317-3.292)	1.000
G-C	0.1665	0.1794	1.091 (0.757-1.574)	0.639
G-T	0.0085	0.0095	1.277 (0.317-5.152)	0.717
FSHR rs6166 A>G /BMP15 rs3810682 C>G				
A-C	0.8223	0.8065	1.000 (reference)	
A-G	0.0027	0.0047	1.286 (0.116-14.26)	1.000
G-C	0.1677	0.1845	1.121 (0.779-1.612)	0.538
G-G	0.0073	0.0043	0.514 (0.06-4.429)	1.000
ESR1 rs9340799 A>G/ESR1 rs2234693 T>C				
A-T	0.7992	0.8076	1.000 (reference)	
A-C	0.1072	0.0935	0.861 (0.537-1.381)	0.535
G-T	0.0058	0.0000	0.276 (0.015-5.145)	0.583
G-C	0.0878	0.0989	1.100 (0.681-1.775)	0.697
ESR1 rs9340799 A>G/BMP15 rs17003221 C>T				
A-C	0.8890	0.8850	1.000 (reference)	
A-T	0.0174	0.0160	0.843 (0.269-2.639)	1.000
G-C	0.0882	0.0916	1.020 (0.626-1.66)	0.938
G-T	0.0054	0.0073	1.264 (0.23-6.949)	0.679

ESR1 rs9340799 A>G/BMP15 rs3810682 C>G				
A-C	0.9009	0.8930	1.000 (reference)	
A-G	0.0055	0.0081	1.272 (0.231-6.993)	0.677
G-C	0.0891	0.0980	1.108 (0.689-1.782)	0.672
G-G	0.0045	0.0009	0.363 (0.019-7.059)	0.563
ESR1 rs2234693 T>C/BMP15 rs17003221 C>T				
T-C	0.7929	0.7935	1.000 (reference)	
T-T	0.0121	0.0140	1.256 (0.374-4.213)	0.750
C-C	0.1842	0.1831	0.993 (0.693-1.423)	0.969
C-T	0.0108	0.0094	0.942 (0.248-3.583)	1.000
ESR1 rs2234693 T>C/BMP15 rs3810682 C>G				
T-C	0.8013	0.8052	1.000 (reference)	
T-G	0.0037	0.0023	0.835 (0.086-8.073)	1.000
C-C	0.1887	0.1858	0.987 (0.691-1.409)	0.941
C-G	0.0063	0.0067	1.252 (0.228-6.888)	1.000
BMP15 rs17003221 C>T/BMP15 rs3810682 C>G				
C-C	0.9676	0.9693	1.000 (reference)	
C-G	0.0095	0.0074	0.719 (0.148-3.485)	1.000
T-C	0.0224	0.0217	0.944 (0.365-2.438)	0.905
T-G	0.0005	0.0016	N/A	N/A

Note: For AOR was adjusted by age.
COR, crude odds ratio; AOR, adjusted odds ratio; 95% CI, 95% confidence interval.

Supplementary Table S2. Differences of various clinical parameters according to gene polymorphisms in total participants.

Genotypes	FSH (mIU/ml) Mean ± SD	LH (mIU/ml) Mean ± SD	E2 (pg/ml) Mean ± SD
***FSHR* rs6165 A>G**			
AA	21.163±25.993	9.851±17.048	25.709±17.821
AG	32.689±29.722	13.749±13.074	23.915±22.435
GG	25.607±28.914	10.435±11.067	34.363±84.215
P	0.057	0.251	0.507
***FSHR* rs6166 A>G**			
AA	22.059±26.444	10.127±16.905	25.903±18.478
AG	31.828±29.634	13.442±13.113	23.455±21.700
GG	26.102±29.165	10.670±11.137	34.818±85.438
P	0.106	0.373	0.468
***ESR1* rs9340799 A>G**			
AA	24.040±26.934	9.564±10.336	27.905±50.597
AG	30.583±29.828	15.514±20.973	23.225±17.664
GG	48.660±41.360	17.040±14.910	47.825±36.124
P	0.295	0.663	0.511
***ESR1* rs2234693 T>C**			
TT	25.268±28.177	14.479±22.852	24.982±19.763
TC	30.907±30.310	12.772±13.356	23.440±18.079
CC	22.603±26.100	8.666±9.587	31.722±64.461
P	0.562	0.219	0.529
***BMP15* rs3810682 C>G**			
CC	26.392±28.181	11.444±14.627	27.128±43.811
CG	30.675±35.229	12.586±12.687	23.567±11.184
GG	N/A	N/A	N/A
P	0.678	0.839	0.843
***BMP15* rs17003221 C>T**			
CC	26.393±28.548	11.503±14.698	27.186±43.609
CT	30.178±26.922	11.196±10.928	23.363±31.792
TT	N/A	N/A	N/A
P	0.698	0.954	0.807

Note: FSH, Follicle Stimulating Hormone; LH, Luteinizing hormone; E2, estradiol

Supplementary Table S3. Differences of various clinical parameters according to gene polymorphisms in control subjects.

Genotypes	FSH Mean ± SD	LH Mean ± SD	E2 Mean ± SD
***FSHR* rs6165 A>G**			
AA	7.985±3.307	3.323±1.887	27.536±14.990
AG	7.359±2.144	4.030±3.834	25.038±14.677
GG	8.982±2.231	3.728±1.555	24.288±12.465
P	0.083	0.441	0.583
***FSHR* rs6166 A>G**			
AA	7.963±3.282	3.309±1.873	27.338±14.927
AG	7.389±2.142	4.022±3.838	25.124±14.631
GG	9.032±2.265	3.794±1.555	24.522±12.691
P	0.082	0.417	0.664
***ESR1* rs9340799 A>G**			
AA	8.116±3.046	3.855±2.843	26.449±15.281
AG	7.834±2.160	3.087±1.598	25.241±12.526
GG	5.700±0.283	1.950±0.495	29.300±0.566
P	0.456	0.247	0.881
***ESR1* rs2234693 T>C**			
TT	8.015±2.348	3.330±1.682	22.050±9.243
TC	7.636±2.529	3.684±2.120	27.123±14.677
CC	8.309±3.219	3.685±3.195	27.027±15.726
P	0.507	0.852	0.37
***BMP15* rs3810682 C>G**			
CC	8.017±2.796	3.615±2.606	26.117±14.561
CG	7.6±3.616	3.725±1.565	26.68±9.146
GG	N/A	N/A	N/A
P	0.747	0.934	0.932
***BMP15* rs17003221 C>T**			
CC	8.003±2.806	3.626±2.626	26.773±14.183
CT	7.92±3.419	3.48±0.823	13.18±11.866
TT	N/A	N/A	N/A
P	0.949	0.902	**0.038**

Note: FSH, Follicle Stimulating Hormone; LH, Luteinizing hormone; E2, estradiol

Supplementary Table S4. Combined genotype analysis for the polymorphisms in POI patients and controls

Genotype combinations	Controls (n=350)	POI patients (n=139)	AOR	*P*
FSHR rs6165 A>G /FSHR rs6166 A>G				
AA/AA	151(43.1)	50(36.0)	1.000(reference)	
AG/AG	141(40.3)	68(48.9)	1.609 (0.946-2.736)	0.079
GG/AG	2(0.6)	1(0.7)	4.290 (0.327-56.296)	0.268
GG/GG	50(14.3)	18(12.9)	1.294 (0.560-2.988)	0.546
FSHR rs6165 A>G / ESR1 rs9340799 A>G				
AA/AA	103(29.4)	28(20.1)	1.000(reference)	
AA/AG	42(12.0)	20(14.4)	1.845 (0.765-4.451)	0.173
AA/GG	6(1.7)	2(1.4)	1.934 (0.195-19.135)	0.573
AG/AA	97(27.7)	48(34.5)	1.957 (0.983-3.895)	0.056
AG/AG	46(13.1)	18(12.9)	1.717 (0.725-4.063)	0.219
AG/GG	4(1.1)	4(2.9)	5.693 (1.088-29.792)	0.039
GG/AA	32(9.1)	14(10.1)	2.163 (0.721-6.485)	0.169
GG/AG	17(4.9)	5(3.6)	1.699 (0.475-6.073)	0.415
FSHR rs6165 A>G / ESR1 rs2234693 T>C				
AA/TT	60(17.1)	17(12.2)	1.000(reference)	
AG/TT	54(15.4)	27(19.4)	1.668 (0.706-3.943)	0.244
AG/TC	67(19.1)	32(23.0)	1.544 (0.679-3.511)	0.300
AG/CC	26(7.4)	11(7.9)	1.878 (0.619-5.699)	0.266
GG/TT	18(5.1)	10(7.2)	2.137 (0.529-8.627)	0.286
GG/CC	7(2.0)	2(1.4)	3.171 (0.457-22.000)	0.243
FSHR rs6165 A>G / BMP15 rs17003221 C>T				
AA/CC	135(38.6)	44(31.7)	1.000(reference)	
AA/CT	16(4.6)	6(4.3)	2.159 (0.718-6.493)	0.171
AG/CC	134(38.3)	65(46.8)	1.874 (1.059-3.316)	0.031
GG/CC	49(14.0)	17(12.2)	1.552 (0.649-3.711)	0.323
FSHR rs6165 A>G / BMP15 rs3810682 C>G				
AA/CC	148(42.3)	48(34.5)	1.000(reference)	
AA/CG	3(0.9)	2(1.4)	6.515 (0.890-47.704)	0.065
AG/CC	138(39.4)	67(48.2)	1.659 (0.961-2.865)	0.069
GG/CC	50(14.3)	19(13.7)	1.512 (0.665-3.439)	0.324
FSHR rs6166 A>G / ESR1 rs9340799 A>G				
AA/AA	108(30.9)	28(20.1)	1.000(reference)	
AA/AG	42(12.0)	22(15.8)	1.915 (0.794-4.617)	0.148
AG/AA	94(26.9)	48(34.5)	2.106 (1.058-4.190)	0.034
AG/AG	45(12.9)	17(12.2)	2.007 (0.861-4.676)	0.107
AG/GG	4(1.1)	4(2.9)	5.940 (1.134-31.131)	0.035
GG/AA	30(8.6)	14(10.1)	2.336 (0.778-7.018)	0.131
FSHR rs6166 A>G / ESR1 rs2234693 T>C				
AA/TT	63(18.0)	17(12.2)	1.000(reference)	
AG/TT	53(15.1)	27(19.4)	1.779 (0.753-4.204)	0.189
AG/TC	65(18.6)	30(21.6)	1.665 (0.733-3.784)	0.224
AG/CC	25(7.1)	12(8.6)	2.331 (0.791-6.872)	0.125
GG/TT	16(4.6)	10(7.2)	2.368 (0.583-9.616)	0.228
FSHR rs6166 A>G / BMP15 rs17003221 C>T				
AA/CC	140(40.0)	46(33.1)	1.000(reference)	
AA/CT	16(4.6)	6(4.3)	2.223 (0.739-6.688)	0.155
AG/CC	130(37.1)	64(46.0)	2.047 (1.159-3.616)	0.014
GG/CC	48(13.7)	16(11.5)	1.423 (0.582-3.479)	0.439
FSHR rs6166 A>G / BMP15 rs3810682 C>G				
AA/CC	153(43.7)	50(36.0)	1.000(reference)	
AA/CG	3(0.9)	2(1.4)	6.727 (0.917-49.325)	0.061
AG/CC	134(38.3)	66(47.5)	1.807 (1.048-3.114)	0.033
GG/CC	49(14.0)	18(12.9)	1.402 (0.604-3.252)	0.432

ESR1 rs9340799 A>G / ESR1 rs2234693 T>C				
AA/TT	126(36.0)	54(38.8)	1.000(reference)	
AA/TC	87(24.9)	29(20.9)	0.679 (0.345-1.337)	0.263
GG/CC	12(3.4)	6(4.3)	1.534 (0.453-5.202)	0.492
ESR1 rs9340799 A>G / BMP15 rs17003221 C>T				
AA/CC	210(60.0)	82(59.0)	1.000(reference)	
AA/CT	22(6.3)	8(5.8)	0.586 (0.167-2.061)	0.405
AG/CT	8(2.3)	5(3.6)	2.878 (0.892-9.289)	0.077
GG/CC	11(3.1)	6(4.3)	1.759 (0.531-5.825)	0.356
ESR1 rs9340799 A>G / BMP15 rs3810682 C>G				
AA/CC	225(64.3)	86(61.9)	1.000(reference)	
AA/CG	7(2.0)	4(2.9)	2.825 (0.677-11.796)	0.154
AG/CC	99(28.3)	42(30.2)	1.274 (0.744-2.181)	0.378
GG/CC	12(3.4)	6(4.3)	1.816 (0.552-5.977)	0.326
ESR1 rs2234693 T>C / BMP15 rs17003221 C>T				
TT/CC	119(34.0)	48(34.5)	1.000(reference)	
TT/CT	13(3.7)	6(4.3)	0.491 (0.102-2.380)	0.377
ESR1 rs2234693 T>C / BMP15 rs3810682 C>G				
TT/CC	128(36.6)	53(38.1)	1.000(reference)	
TC/CG	8(2.3)	4(2.9)	2.034 (0.568-7.280)	0.275

Note: For AOR was adjusted by age.
AOR, adjusted odds ratio; 95% CI, 95% confidence interval.
