# Supplementary material for: Association of Polymorphisms in FSHR, ESR1, and BMP15 with Primary Ovarian Insufficiency and Meta-Analysis
Source: Diagnostics (Basel). 2024 Aug 28;14(17):1889. doi: 10.3390/diagnostics14171889 (PMC11393966; doi:10.3390/diagnostics14171889)
Supplement: Supplementary file 1 [file diagnostics-14-01889-s001.zip › Supplementary Figures.pdf]

Meta analysis of FSHR polymorphism rs6166 AA VS GA+GG

Study	Patients	Controls	Odds ratio (95% CI)
Cordts 2015	82/96	102/123	1.206 (0.578 - 2.518)
Du 2010	21/37	50/90	1.05 (0.485 - 2.272)
Kim 2011	60/83	109/176	1.604 (0.908 - 2.832)
Ma 2015	32/63	26/58	1.27 (0.621 - 2.598)
Tong 2001	11/16	145/236	1.381 (0.465 - 4.103)
Woad 2013	51/80	54/80	0.847 (0.441 - 1.627)
present study	87/139	194/350	1.345 (0.899 - 2.013)
Total (fixed effects)	344/514	680/1113	1.257 (0.993 - 1.590)
Total (random effects)	344/514	680/1113	1.255 (0.991 - 1.589)
	Significance level		P = 0.8720
	**I^2 (inconsistency)**		0.00 %
	95% CI for I^2		0.00 - 30.21

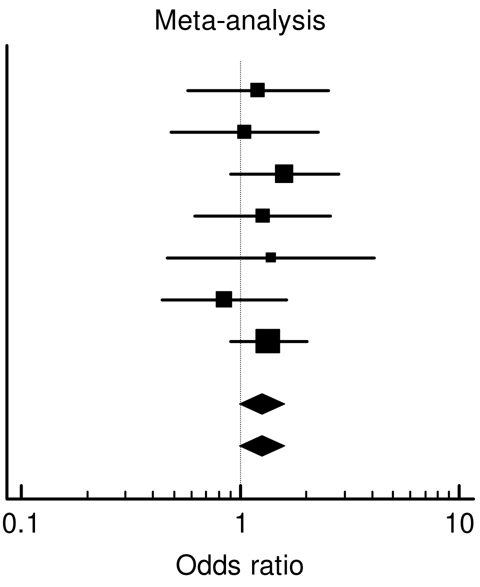

Supplementary Figure S1. Forest plot of meta-analysis of FSHR polymorphisms. Meta-analysis of FSHR rs6166 AA vs GA+GG in POI risk [3,23,25,26,29,31].

Meta analysis of BMP15 polymorphism rs3810682 CC vs CG+GG

Study	Patients	Controls	Odds ratio (95% CI)
Santos 2019	62/70	81/88	0.67 (0.230 - 1.947)
present study	5/139	14/350	0.896 (0.316 - 2.535)
Total (fixed effects)	67/209	95/438	0.779 (0.368 - 1.649)
Total (random effects)	67/209	95/438	0.777 (0.369 - 1.637)
	Significance level		P = 0.7024
	**I^2 (inconsistency)**		0.00 %
	95% CI for I^2		0.00 - 0.00

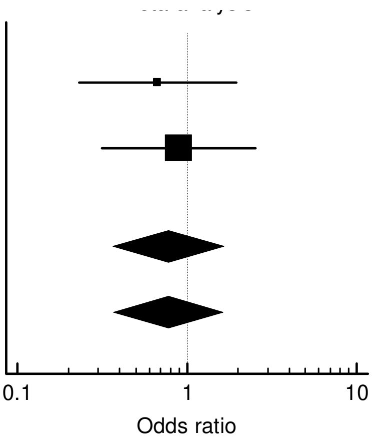

Supplementary Figure S2. Forest plot of meta-analysis of ESR1 polymorphisms. Meta-analysis of rs9340799 AA vs AG+GG (A) and rs2234693 AA vs AG+GG (B) in POI risk [35].
